# LincRNAs *MONC* and *MIR100HG* act as oncogenes in acute megakaryoblastic leukemia

**DOI:** 10.1186/1476-4598-13-171

**Published:** 2014-07-15

**Authors:** Stephan Emmrich, Alexandra Streltsov, Franziska Schmidt, Veera Raghavan Thangapandi, Dirk Reinhardt, Jan-Henning Klusmann

**Affiliations:** 1 Pediatric Hematology and Oncology, Hannover Medical School, Carl-Neuberg-Straße 1, 30625 Hannover, Germany

## Abstract

**Background:**

Long non-coding RNAs (lncRNAs) are recognized as pivotal players during developmental ontogenesis and pathogenesis of cancer. The intronic microRNA (miRNA) clusters *miR-99a ~ 125b-2* and *miR-100 ~ 125b-1* promote progression of acute megakaryoblastic leukemia (AMKL), an aggressive form of hematologic cancers. The function of the lncRNA hostgenes *MIR99AHG* (alias *MONC*) and *MIR100HG* within this ncRNA ensemble remained elusive.

**Results:**

Here we report that lncRNAs *MONC* and *MIR100HG* are highly expressed in AMKL blasts. The transcripts were mainly localized in the nucleus and their expression correlated with the corresponding miRNA clusters. Knockdown of *MONC* or *MIR100HG* impeded leukemic growth of AMKL cell lines and primary patient samples. The development of a lentiviral lncRNA vector to ectopically express lncRNAs without perturbing their secondary structure due to improper termination of the viral transcript, allowed us to study the function of *MONC* independent of the miRNAs in cord blood hematopoietic stem and progenitor cells (HSPCs). We could show that *MONC* interfered with hematopoietic lineage decisions and enhanced the proliferation of immature erythroid progenitor cells.

**Conclusions:**

Our study reveals an unprecedented function of lncRNAs *MONC* and *MIR100HG* as regulators of hematopoiesis and oncogenes in the development of myeloid leukemia.

## Background

It has become apparent that the vast majority of the eukaryotic genome underlies prevalent transcription [1]. Both DNA strands are pervasively transcribed, giving rise to numerous different classes of non-coding RNAs (ncRNAs), including long intergenic RNAs (lincRNAs), antisense RNAs and enhancer RNAs (eRNAs) [2]. This abundant mixture of long (> 200 nt) and short (< 200 nt) non-coding RNAs was misapprehended in the past as transcriptional noise or junk. However, accumulating evidence suggested that transcription factors and other global regulators are prevalent targets of ncRNAs [3]. Thereby, ncRNAs induce changes in histone marks and gene expression *in cis* and *in trans*. For example, *XIST* is crucial for random inactivation of the X chromosome [4]. Beyond that, *Xist* RNA acts as a suppressor of hematologic cancer [5]. Deletion of *Xist* results in the development of a highly aggressive myeloproliferative neoplasm and myelodysplastic syndrome. In contrast, *HOTAIR* regulates expression of the *HOXD* gene family as well as other genes throughout the genome via re-targeting of Polycomb repressive complex 2 (PRC2) [6,7]. Enforced expression of *HOTAIR* in epithelial cancer cells leads to altered histone H3 lysine 27 methylation, gene expression, and increased cancer invasiveness and metastasis. Similarly, *HOTTIP* affects expression of the *HOXA* gene family [8]. Recently, E2F1 transcription factor has been shown to activate lncRNA *ERIC*, which restricts E2F-induced apoptosis during cell cycle progression [9].

**Figure 1:**
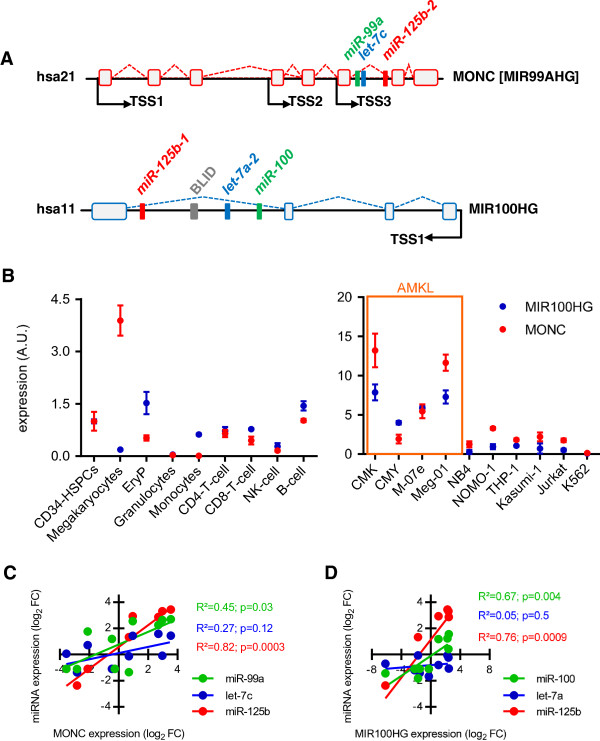
***MiR-99a/100 ~ 125b *cluster host genes in hematopoietic cells and leukemia. A)** Genomic architecture of the *miR-99a/let-7c/miR-125b-2* (hsa21) and *miR-100/let-7a-2/miR-125b-1* (hsa11) cluster. *MIR99AHG* (alias *MONC*) and *MIR100HG* represent the lincRNA host genes of the miRNA cluster, TSSs were determined by 5’RACE-PCR [15]. **B)** Transcript quantification of *MONC* and *MIR100HG* by qRT-PCR in sorted CD34^+^ HSPCs, CD36^+^/ GlyA^+^ erythroid cells, CD41^+^/ CD42b^+^ megakaryocytes, CD15^+^/ CD66b^+^ neutrophil granulocytes, CD14^+^ monocytes, CD3^+^/ CD4^+^/ CD8^−^ and CD3^+^/ CD4^−^/ CD8^+^ T-cells, respectively, CD56^+^/ CD3^−^ NK cells and CD19^+^/ CD3^−^/ CD56^−^ B-cells (*left panel*; n = 5 each) as well as indicated cell lines (*right panel*). The *B2M* gene was used as reference; A.U., arbitrary units. **C)** Correlation plots and statistics of *MONC* and **D)***MIR100HG* expression with their cluster miRNA expression in NB4, NOMO-1, THP-1, Kasumi-1, Jurkat, K562, M-07e, Meg-01, CMK and CMY cells measured by qRT-PCR. **(B-D)** Data are presented as mean ± s.d.

Acute myeloid leukemia (AML) is an aggressive form of hematologic cancers with a 5-year overall survival between 30 and 40% in adults [10]. While AML is generally less common in children, inherited molecular lesions can cause a genetic background, which predisposes to malignant transformation and AML. Particularly children with Down syndrome (DS), i.e. trisomy 21, have a 400-fold increased risk [11] to develop acute megakaryoblastic leukemia (AMKL). Patients with DS-AMKL have an excellent prognosis with 5-year overall survival rates of about 80%, while non-DS-AMKL patients have poor survival rates of only 14% to 34% despite high intensity chemotherapy [12,13]. The molecular mechanisms underlying this AML subtype remain incompletely understood. We recently reported the characterization of an oncogenic microRNA (miRNA) on chromosome 21 (hsa21), *miR-125b-2*, which is highly expressed in DS-AMKL and non-DS-AMKL. *miR-125b-2* increased proliferation and self-renewal of human and mouse megakaryocytic progenitors (MPs) and megakaryocytic/erythroid progenitors (MEPs) [14]. This small RNA is located in a phylogenetically conserved ncRNA ensemble, consisting of two other miRNAs (*miR-99a* and *let-7c*) and the lncRNA hostgene MIR99AHG, which we termed megakaryocytic oncogenic non-coding RNA (*MONC*) (Figure 1A). A homolog of the *miR-99a ~ 125b-2* polycistron on hsa21 can be found in identical configuration in the intron of the lincRNA *MIR100HG* on hsa11 (*miR-100 ~ 125b-1*). We could previously demonstrate that *miR-100 ~ 125b-1* and *miR-99a ~ 125b-2* protect megakaryoblasts and leukemic cells from TGFβ1-mediated proliferation arrest and apoptosis [15]. However, the role of the lncRNA hostgenes in this ncRNA ensemble remained elusive.

In the present study, we characterized the function of *MONC* and *MIR100HG* and demonstrate an unprecedented role of lncRNAs *MONC* and *MIR100HG* during hematopoiesis and the pathogenesis of AMKL.

## Results

### *MiR-99a/100 ~ 125b* cluster lincRNAs are overexpressed in AMKL

The *miR-99a/100 ~ 125b* clusters on hsa11 and hsa21 are central regulators of stem cell homeostasis and leukemogenesis and are hosted in introns of *MIR100HG* and *MONC*, respectively. We mapped the transcriptional start sites (TSS) of both clusters by 5’RACE-PCR and demonstrated that the miRNAs are transcribed as one polycistronic transcript together with their host genes [15]. qRT-PCR expression profiling of spliced *MONC* and *MIR100HG* trancripts throughout hematopoietic lineages demonstrated higher expression of *MONC* in megakaryocytes, HSPCs and B-cells and higher expression of *MIR100HG* in erythroid cells, HSPCs and B-cells as compared to the other blood lineages (Figure 1B). Furthermore, *MONC* and *MIR100HG* are higher expressed in AMKL cell lines compared to various other leukemic cell lines (Figure 1B). Regression analysis confirmed positive correlation of *MONC* and *MIR100HG* with their respective miRNA polycistrons (Figure 1C,D). However, both mature *let-7* isoforms did not show a strong positive correlation with their lincRNA host genes, suggesting active LIN28- and/or *miR-107*-mediated suppression of let-7 in *MONC*- and *MIR100HG*-high expressing cells [16,17].

Thus, expression patterns of spliced *MONC* and *MIR100HG* transcripts implicate an independent, yet unknown function in hematopoietic regulation and transformation.

### Knockdown of *MIR100HG* impairs cell proliferation and viability

Therefore, we investigated the consequences of *MIR100HG* knockdown in the AMKL cell line Meg-01 with a high endogenous expression (Figure 1B). To achieve sufficient knockdown of endogenous *MIR100HG,* we designed two different shRNAs and verified a knockdown efficiency of 65% for sh-*MIR100HG* #1 and 80% for sh-*MIR100HG* #2 by qRT-PCR (Additional file 1: Figure S1A).

**Figure 2:**
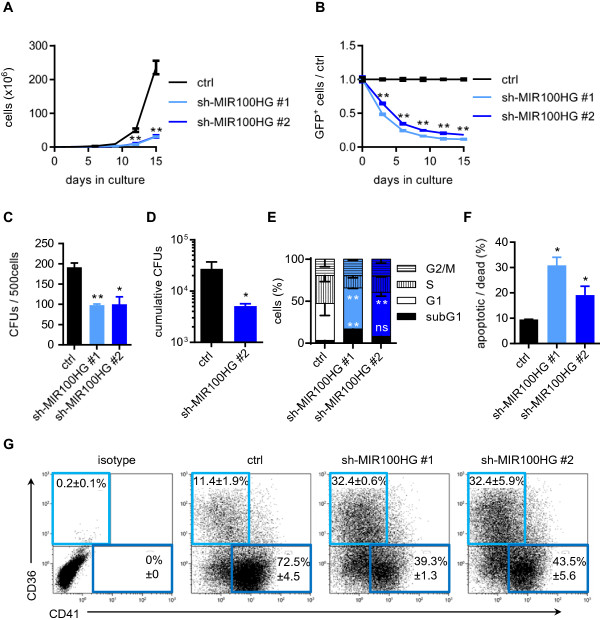
**Knockdown of *MIR100HG *confers growth disadvantage to AMKL cells. A)** Number of shRNA- or ctrl-transduced Meg-01 cells (n = 2). **B)** Fraction of Cerulean^+^ shRNA-transduced cells at indicated time points of culture is shown in relation to the ctrl construct (n = 2; Two-way ANOVA was performed to compare the mean of each construct at each time point to ctrl). **C)** Number of colonies from methylcellulose-based CFU assays of shRNA-transduced Meg-01 cells (n = 2). **D)** Cumulative number of CFUs after one round of replating of sh-*MIR100HG* #2 in Meg-01 cells (n = 2). **E)** Percentage of shRNA-transduced Meg-01 cells in subG1 (BrdU^−^/7-AAD^−^), G1 (BrdU^low^/7-AAD^low/high^), S-phase (BrdU^+^/7-AAD^low/high^) and G2/M fraction (BrdU^low^/7-AAD^high^) (n = 2). Asterisks are indicated for subG1 and S phases. **F)** Percentage of apoptotic/dead (Annexin V^+^) shRNA-transduced Meg-01 cells after 5 days of culture (n = 2). **G)** Representative density plots of viable, Cerulean^+^ Meg-01 cells for indicated surface markers as measured by flow cytometry after 5 days of culture (n = 4). **(A-G)** Data are presented as mean ± s.d. *P < 0.05; **P < 0.01.

Proliferation of Meg-01 cells was impaired upon *MIR100HG* knockdown (Figure 2A). In competition assays, where sh-*MIR100HG*-transduced Cerulean-positive (Cer^+^) Meg-01 cells were mixed with non-silencing control shRNA-transduced mCherry-positive (mCh^+^) Meg-01 cells, both shRNAs against *MIR100HG* conferred a strong growth disadvantage (Figure 2B). In contrast, proliferation of K562 cells with low to absent endogenous *MIR100HG* expression was mainly unaffected by sh-*MIR100HG-*transduction (Additional file 1: Figure S1B-C). The colony-forming capacity of Meg-01 cells was decreased upon *MIR100HG*-knockdown (Figure 2C). This effect was even aggravated in replating experiments for sh-*MIR100HG* #2, the construct with the stronger knockdown efficacy (Figure 2D). In BrdU cell cycle analyses of Meg-01 cells, we observed an increase in the apoptotic subG1 fraction accompanied by a decrease of cycling cells in S phase upon *MIR100HG* knockdown (Figure 2E). Accordingly, we monitored a significant increase of Annexin^+^ apoptotic cells (Figure 2 F). Interestingly, *MIR100HG* knockdown changed the surface marker expression on the leukemic megakaryoblasts (Figure 2G). While the percentage of CD36^+^ cells increased from 11% in controls to 32%, the percentage of CD41^+^ cells was ~1.8-fold reduced.

Taken together, knockdown of *MIR100HG* impaired cell viability and replating-efficiency of AMKL cells, while changing lineage surface marker expression.

### Knockdown of *MONC* reduces cell proliferation and viability

*MONC* is encoded on hsa21 and highly upregulated in both DS-AMKL (trisomy 21) and non-DS-AMKL cell lines (Figure 1B). Therefore we sought to evaluate the consequences of *MONC* knockdown in CMK and Meg-01 cell lines, representing those two entities. As a control, we used K562 cells with low to absent *MONC* expression (Figure 1B). We designed a total of 8 different shRNAs covering different sites of *MONC*. Only one shRNA had sufficient knockdown efficacy (Additional file 2: Figure S2A).

**Figure 3:**
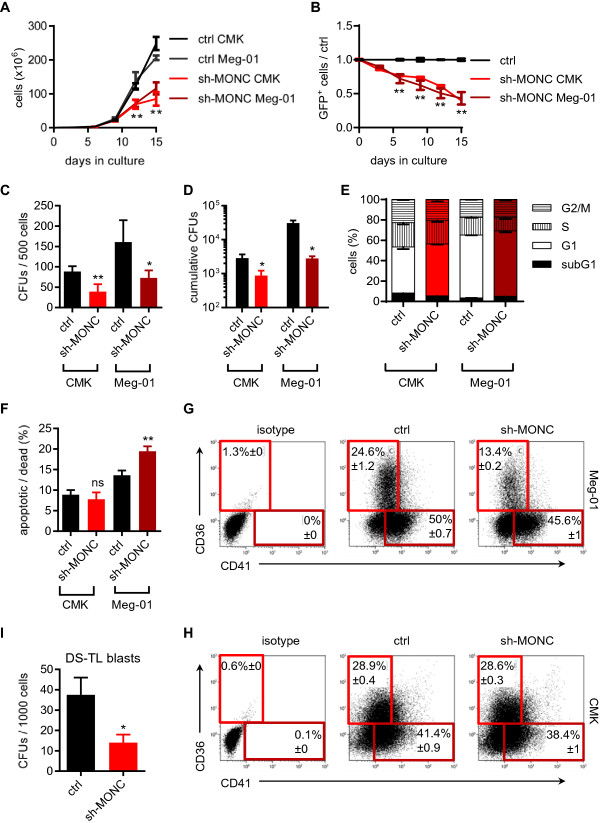
**Knockdown of *MONC *reduces proliferation and viability of AMKL cells. A)** Number of shRNA- or ctrl-transduced CMK and Meg-01 cells (n = 3). **B)** Fraction of Cerulean^+^ shRNA-transduced cells at indicated time points of culture is shown in relation to the ctrl construct (n = 3; Two-way ANOVA was performed to compare the mean of each construct at each time point to ctrl). **C)** Number of colonies from methylcellulose-based CFU assays of indicated shRNA-transduced CMK and Meg-01 cells (n = 3). **D)** Cumulative number of CFUs after one round of replating of sh-*MONC* in CMK and Meg-01 cells (n = 3). **E)** Percentage of shRNA-transduced Meg-01 cells in subG1 (BrdU^−^/7-AAD^−^), G1 (BrdU^low^/7-AAD^low/high^), S-phase (BrdU^+^/7-AAD^low/high^) and G2/M fraction (BrdU^low^/7-AAD^high^) (n = 3). **F)** Percentage of apoptotic (Annexin V^+^/7-AAD^−^) and dead (7-AAD^+^) cells for shRNA-transduced CMK and Meg-01 cells measured by flow cytometry after 5 days of culture (n = 3). **G-H)** Merged density plots of viable, Cerulean^+^**G)** Meg-01 and **H)** CMK cells for indicated surface markers as measured by flow cytometry after 5 days of culture (n = 4); population frequencies with errors are displayed for respective framed gates. **I)** Number of colonies from methylcellulose-based CFU assays of indicated shRNA-transduced Down-Syndrome transient leukemia blasts (n = 2; error bars show variation). **(A-H)** Data are presented as mean ± s.d. *P < 0.05; **P < 0.01.

Cell proliferation was impaired by *MONC*-knockdown in AMKL cells, yet was unaffected in K562 cells (Figure 3A, Additional file 2: Figure S2B). In growth competition assays we noticed a strong decline of Cer^+^ sh-*MONC*-transduced AMKL cells (Figure 3B). Similarly, monitoring of cell growth by automated microscopy in K562, CMK and M-07 cell lines showed a reduction of sh-*MONC*-transduced AMKL cells, whereas their number was insignificantly changed in K562 cells (Additional file 2: Figure S2C). Accordingly, the colony-forming capacity of sh-*MONC*-transduced Meg-01 and CMK cells -but not K562 cells- was reduced (Figure 3C, Additional file 2: Figure S2D). Also replating experiments showed a decrease in the cumulative CFU number for both AMKL cell lines (Figure 3D). Cell cycle analysis demonstrated insignificant changes upon *MONC* knockdown (Figure 3E). sh-*MONC* favored apoptosis in Meg-01 but not in CMK cells as measured by Annexin V staining (Figure 3F). Quantification of megakaryocytic-erythroid surface markers (CD41 and CD36) revealed a reduction of CD36^+^ Meg-01 cells upon *MONC* knockdown (Figure 3G,H), while this effect was not observed in CMK cells.

Experiments in primary AML cells are challenging. However, they are pertinent to extrapolate observation made in cell lines to the situation in vivo. Strikingly, when DS transient leukemia (DS-TL) blasts were transduced with sh-*MONC*, colony-forming capacity was diminished (Figure 3I), implicating a role of hsa21-encoded *MONC* in the development and maintenance of trisomy 21-associated leukemia.

### Design and cloning of a lentiviral lincRNA expression vector

To expand our knowledge about *MONC* in hematopoietic cells, we sought to ectopically express the lincRNA in CD34^+^-HSPCs from healthy donors. Expression of the spliced lincRNA would also allow us to dissect its function from the intronic miRNAs. However, there are several challenges to consider. The transfection efficiency of plasmid DNA or RNA into CD34^+^-HSPCs is very low [18]. Furthermore, transfected nucleic acids are diluted out during cell divisions. Thus, an integrating lentiviral vector stably overexpressing the transgene and a selection marker is advantageous. However, the lincRNA transcript from the lentiviral vector should be equivalent to the endogenous lincRNA. Transcription of adjacent proviral DNA due to improper termination downstream of the lincRNA transcript could alter the secondary structure of the lincRNA and therewith its function [19]. Thus, conventional vectors that are used for expression of protein coding genes are not suitable for studying the function of lncRNAs.

**Figure 4:**
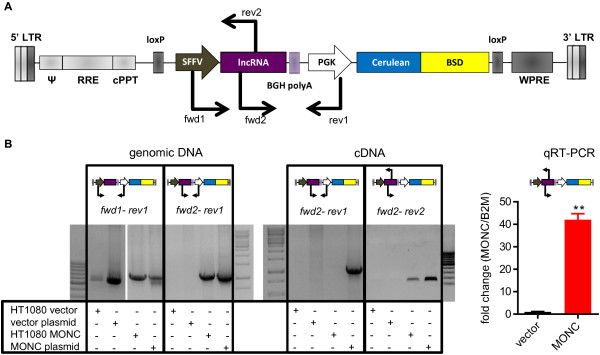
**Design and evaluation of a lentiviral lncRNA overexpression vector. A)** Schematic vector map. PCR primers used in **B)** are indicated. **B)** DNA electrophoresis gel of control PCRs validating the lncRNA expression and termination. Primer pair fwd1-rev1 demonstrates genomic integration of transduced cells, pair fwd2-rev1 indicates functional PolyA signal upon product absence in cDNA samples, and pair fwd2-rev2 detects specifically the *MONC* transcript. For all primer combinations plasmid DNA (vector plasmid, *MONC* plasmid) was used as a control for respective genomic or cDNA samples. Vector, empty LeGO-CeB/lnc. right graph: qRT-PCR quantification of *MONC* in transduced HT1080 cells (Data are presented as mean ± s.d. *P < 0.05; **P < 0.01).

Therefore, we modified the widely used LeGO-CeB vector [20] by removing the murine U6 expression cassette for small RNAs and inserting a bovine growth hormone polyadenylation signal (BGH polyA) followed by the phosphoglycerate kinase (PGK) promoter. This created the LeGO-CeB/lnc vector, featuring a spleen focus-forming virus promoter (SFFV)-driven lincRNA expression cassette terminated by a polyA signal, and an independent PGK-driven marker cassette (Figure 4A). Although an in-sense oriented polyA signal interferes with viral genome RNA replication resulting in generally low titer yields, infective viral particles are generated in sufficient amounts to transduce primary cells as outlined below.

Spliced *MIR100HG* RNA has a length of 3082 nt (NR_024430.1), precluding its cloning and evaluation with the described lentiviral vector. *MONC* has a length of 710 nt (ENST00000445461) (Additional file 3: Figure S3), which allowed successful cloning and production of functional lentiviral particles. Using genomic DNA (gDNA) of LeGO-CeB/lnc:*MONC* and LeGO-CeB/lnc:empty (vector) transduced HT1080 cells, we could confirm genomic integration of both vectors by PCR (Figure 4B, left gel charts). PCR using a forward primer (fwd2) binding to the *MONC* insert and a reverse primer binding to the downstream PGK promoter (rev1) validated the presence of *MONC* proviral DNA in the genome of *MONC*-transduced cells only (Figure 4B, left gel charts). RT-PCR with the same primer pair on cDNA of transduced HT1080 cells could not detect a corresponding transcript. In contrast, RT-PCR with a primer pair binding to MONC detected expression of the transgene, demonstrating that transcription of the lincRNA from the SFFV promoter was efficiently terminated by the polyA signal before the PGK promoter. qRT-PCR showed 40-fold upregulation of *MONC* expression in LeGO-CeB/lnc-*MONC*-transduced HT1080 cells (Figure 4B, right graph). Hence, we engineered a lentiviral lncRNA expression vector, LeGO-CeB/lnc, which was validated to produce integration-competent virus and to express the lncRNA insert without vector-derived RNA.

### Ectopic *MONC* interferes with myeloid differentiation of HSPCs

**Figure 5:**
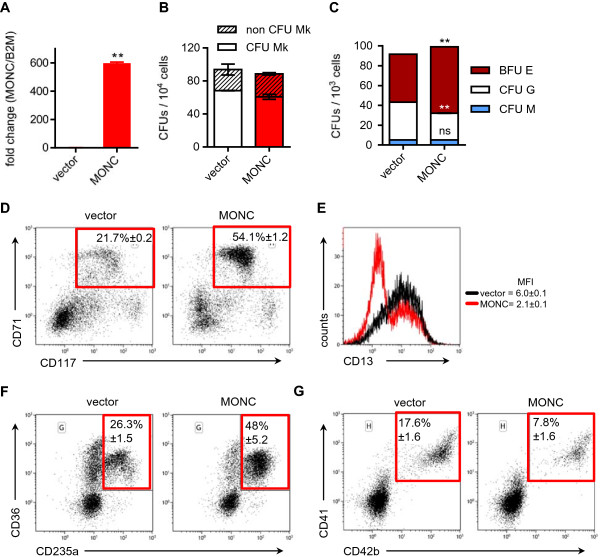
***MONC *leads the expansion of immature erythroid precursors. A)** qRT-PCR of *MONC*-transduced CD34^+^-HSPCs. **B)** Numbers of megakaryocytic (CD41^+^) and non-megakaryocytic (CD41^−^) CFUs from Megacult® assays of transduced HSPCs after 14 days (n = 2). **C)** Number of colonies in the methocellulose-based CFU-assay of transduced HSPCs after 12 days (n = 2). **D)** Representative flow cytometry dot plots of transduced HSPCs stained for CD71 and CD117 on day 4 of in vitro culture (n = 2). **E)** Representative flow cytometry histogram of CD13-stained transduced HSPCs on day 4 of in vitro culture (n = 2). Mean fluorescence intensities (MFI) are indicated. **F-G)** Representative flow cytometry dot plots of transduced HSPCs stained for **F)** CD36 and CD235a and **G)** stained for CD41 and CD42b after 7 days of mixed erythroid-megakaryocytic differentiation culture (n = 2); population frequencies with errors are displayed for respective framed gates. **(A-G)** Data are presented as mean ± s.d. *P < 0.05; **P < 0.01.

Next we overexpressed *MONC* in cord-blood (CB) CD34^+^-HSPCs to determine its impact on hematopoietic lineage decisions. qRT-PCR in transduced HSPCs revealed more than 500-fold increased *MONC* levels (Figure 5A). This expression levels are comparable with the leukemic setting, as *MONC* levels are ~450-fold elevated in CMK cells compared to CD34^+^-HSPCs (Additional file 4: Figure S4). In CFU-megakaryocyte (CFU-MK) assays, the number of colonies was slightly reduced upon ectopic *MONC* expression (Figure 5B). Concordantly, in methocellulose-based myeloid CFU-assays *MONC* led to a decrease of granulocytic CFU-Gs, while erythroid BFU-Es were expanded (Figure 5C). However, in both CFU assays the total number of colonies was not significantly changed. Interestingly, culturing of HSPCs in a growth medium promoting multilineage progenitor expansion resulted in a more than 2-fold increase of CD117^+^/CD71^+^ erythroid progenitor cells by *MONC* (Figure 5D). Strikingly, the percentage of CD13^+^ myelomonocytic progenitors was strongly reduced by *MONC* (Figure 5E). In liquid cultures promoting megakaryocytic and erythroid differentiation, we noted a switch in lineage decision. This was evident by a sharp increase of CD36^+^/CD235a^+^ erythroid cells (Figure 5F) and decrease of CD41^+^/CD42b^+^ megakaryocytes (Figure 5G). These data are in concordance with the BFU-E expansion and CFU-MK reduction in the CFU-assays.

In conclusion, enforced *MONC* expression in normal HSPCs changes the lineage bias towards the erythroid compartment and leads to the expansion of immature erythroid progenitor cells.

### *MONC* and *MIR100HG* are located in the nucleus

**Figure 6:**
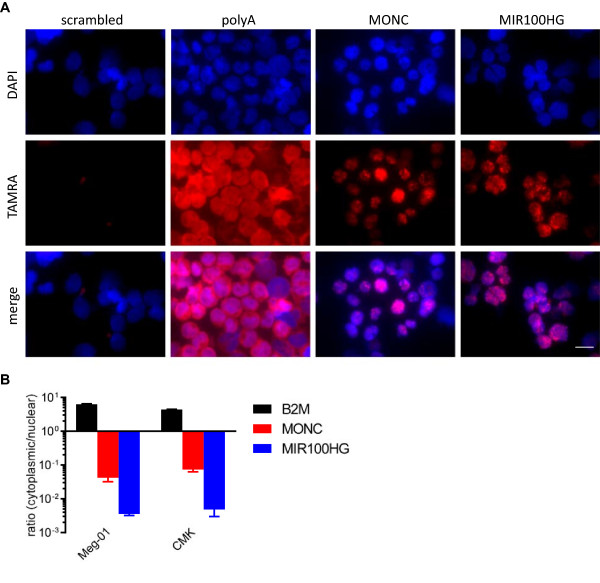
**Subcellular localization of *MONC *and *MIR100HG. *A)** RNA-FISH with LNA-probes for *MONC* and *MIR100HG* transcripts in CMK cells (n = 3). A scrambled oligo was used as negative control and a probe against poly-adenylated transcripts as a positive control (scale bars: 10 μm). **B)** qRT-PR for *MONC* and *MIR100HG* in fractionated RNA, showing a < 1 ratio. Cytoplasmic B2M RNA with a > 1 ratio is shown as control.

To determine the subcellular localization, we applied RNA fluorescence in situ hybridization (RNA-FISH) to capture endogenous *MONC* and *MIR100HG* signals by locked nucleic acids (LNA) probes in CMK cells. Both *MONC* and *MIR100HG* probes showed predominantly a textured staining of nuclear areas (Figure 6A), as compared to polyadenylated mRNA (positive control). To confirm this localization pattern by an alternative method, we applied subcellular RNA fractionation followed by qRT-PCR to calculate a cytoplasma:nucleus ratio. As expected beta-2 microglobulin (B2M) mRNA showed a clear cytoplasmic localization, while both *MONC* and *MIR100HG* transcripts showed a strong prevalence for nuclear localization (Figure 6B).

## Discussion

Here we show the predominant expression of lincRNAs *MIR100HG* and *MONC* in HSPCs and erytroid/megakaryocytic cells and their dysregulation in megakaryoblastic leukemia. The growth of AMKL cells was dependent on the continuous expression of both lincRNAs. Enforced expression of spliced *MONC* in normal HSPCs led to the predominant differentiation along the erythroid lineage and expansion of CD117^+^/CD71^+^ immature erythroid progenitor cells at the expense of myeloid and megakaryocytic differentiation. Favoring fast growing progenitor stages by *MONC* might therefore provide a context for malignant transformation. Thus, it seems unlikely that those lincRNAs act merely as host genes or byproducts of *miR-99a/100 ~ 125b* cluster transcription by providing a Polymerase II promoter, as exemplified for the *miR-31* locus in breast cancer [21].

Results of RNA-FISH and qRT-PCR on fractionated RNA pointed towards a nuclear localization of *MONC* and *MIR100HG*. Recently an interesting hypothesis regarding the biological function of lncRNAs suggested that lncRNAs serve as subcellular address codes for other biomolecules [22]. Especially the nucleus with its higher order structures is an organelle suitable for lncRNA-directed spatial organization. This is particularly reflected by several lncRNAs interacting with chromatin remodelers to recruit them to specific genomic loci or subnuclear sites. E.g., Air mediates silencing of paternal alleles of multiple genes, Xist controls inactivation of one X chromosome in females, and Kcnq1ot1 regulates imprinting of placental genes. All three lncRNAs act by allele-specific directing of PRC2 or G9a, thereby leading to histone methylation of H3K27me3 or H3K9me3 [23-25]. Meanwhile, a compelling discovery in Drosophila unravelled the distinction of five principal chromatin types by protein components, which form separate domains [26]. Recently this model became complemented by computational analysis of genome-wide epigenetic marks distribution to 4 principal chromatin types with virtually identical classification [27]. With this the genome can be compared with the design of a roadmap, where districts are defined by chromatin-bound proteins and epigenetic marks, lncRNAs form the street names and the gene loci regulatory sequences represent the house numbers. The fluorescence signals for both lincRNA probes show a broad, irregular dispersion rather than singular site distribution over the nucleus. This may indicate a contribution of either host gene to a ternary chromatin modifying or remodeling complex acting at multiple nuclear domains. The chromatin modifying SWI/SNF complex subunit BRG1 is associated with melanoma progression [28]. However, the lncRNA SChLAP1 imparts functioning of SWI/SNF complexes contributing to development of lethal prostate cancers [29]. Repression of the tumor suppressor INK4b-ARF-INK4a locus by ANRIL lncRNA is mediated by both Polycomb repressive complex-1 (PRC1) and PRC2, increasing the likelihood of oncogenesis [30]. Meanwhile a WDR5 mutant defective in RNA binding fails to activate gene expression in embryonic stem cells by the Trithorax Group/Mixed-Lineage-Leukemia complex [31]. Further research identifying protein interaction partners and pinpointing precise subnuclear areas and DNA target sequences of *MONC* and *MIR100HG* is warranted.

## Conclusions

This study characterizes for the first time lincRNAs during megakaryopoiesis and AMKL. *MONC* and *MIR100HG*, the human *miR-99a/100 ~ 125b* cluster host genes, reside in the nuclear cell compartment, where they play a role in the regulation of erythro-megakaryocytic development. In AMKL they contribute to the maintenance of leukemic growth. Given the central role of *miR-99 ~ 125* polycistron miRNAs in AML, advanced understanding of the gene products from these loci will ultimately lead to therapy improvements of this aggressive malignancy.

## Methods

### Patient samples and cell lines

The AML-‘Berlin-Frankfurt-Münster’ Study Group (AML-BFM-SG, Hannover, Germany) provided all patient samples. CB HSPCs from donors were positively selected by labeling CD34 expressing cells with magnetic cell-sorting beads (Miltenyi Biotech). Culture conditions for maintenance, megakaryocytic or megakaryocytic/erythroid in vitro differentiation of CD34^+^-HSPCs were described elsewhere [32-34]. Cell lines (CMK, Meg-01, K562, HT1080 and 293T) were purchased from the German National Resource Center for Biological Material (DSMZ) and maintained under recommended conditions. All investigations had been approved by the local Ethics Committee.

### Constructs and lentivirus

Cloning of shRNAs into a modified LeGO vector was performed as previously described [32,35]. A non-silencing shRNA in the *miR-30* backbone (Open Biosystems) was subcloned to the LeGO vector and used as control (referred to as non-silencing miRNA). ShRNAs against human *MONC* were obtained from Open Biosystems (Clone IDs V2LHS_206411, V2LHS_208623) or designed by TRC (http://www.broadinstitute.org/rnai/public/) and subcloned into the LeGO *miR-30* backbone construct. Stable lincRNA overexpression was achieved using a novel modified LeGO vector, LeGO-CeB/lnc. Briefly, we removed the murine U6 promoter by *XhoI* and *XbaI* digestion with subsequent end filling by a proof reading polymerase (Phusion II, Finnzymes) and religation. Next we inserted the PGK promoter from pMSCV-Puro-IRES-GFP [36] retrovector with a 5’ 20 nt spacer containing *NsiI* site into the *BamHI* site adjacent to the SFFV promoter. A BGH polyA signal from pMIRREPORT was inserted into *NsiI*. An oligo with the MCS for lncRNA fragments was inserted via NotI between SFFV and polyA. The *MONC* isoform MIR99AHG-iso6 (ENST00000445461) was synthesized by GeneArt (lifetech). Lentiviral supernatant was generated and collected using standard protocols as described [32].

### Transduction and hematopoietic assays

CD34+ HSPCs were lentivirally transduced on RetroNectin-coated (Takara) plates as described [32]. Methylcellulose-based (Methylcellulose Base and Complete, RnD Systems) and collagen-based (Megacult®, Stem Cell Technologies) colony-forming assays were carried out according to the manufacturers’ instructions. Serial replating was performed as described previously [33]. Cumulative colony numbers were calculated with the following equation: $\mathrm{CFU}\left(k\right)=\sum_{n=1}^{k}{\mathrm{CFU}}_{n}$, where CFU_n_ = number of counted colonies from respective platings (n). Note that if a fraction of cells (f) from the 1^st^ plating was replated for the 2^nd^ plating, then $\mathrm{CFU}\left(k\right)={\mathrm{CFU}}_{1}+\frac{{\mathrm{CFU}}_{2}}{f_{1}}$.

### Cell growth, cell cycle and apoptosis assays

Apoptosis was detected with the Annexin V Apoptosis Detection Kit II (Becton Dickinson) and cell cycle was analyzed with the the BrDU Flow Kit (Becton Dickinson). All assays were performed according to the manufacturer’s instructions. Growth competition assays were performed by mixing each transduced Cerulean + population 1:1 with a control population expressing eGFP.

### Flow Cytometry and Cell Sorting

Transduced HSPCs were sorted based on GFP-expression. Flow Cytometry was performed on a Navios 10/3 (Beckman Coulter). Kaluza 1.2 (Beckman Coulter) was used for data analysis. Staining and measuring were performed according to standard protocols and as described previously using the antibodies PE-CD42b, PC5.5-CD13, PC7-CD41, PC7-CD117, AlexaFluor®750-CD235a (all Beckman Coulter), APC-CD36, APC-CD42b (both Becton Dickinson) and PacificBlue-CD71 (Exbio) [14].

### RNA isolation and Quantitative real-time PCR (qRT-PCR)

Standard RNA isolation, cDNA synthesis and mRNA qRT-PCR were done as described [14]. qRT-PCR primer sequences are available upon request, B2M was used as reference gene. MiRNA-Detection was performed with TaqMan miRNA assays (ABI), RNU44 was used as reference gene. All data were analyzed in a StepOnePlus Cycler (ABI) using the geNORM ΔΔCt equations. RNA fractionation into cytoplasmic and nuclear lysates was done by PARIS Kit (Ambion, lifetech) according to manufacturers’ instructions.

### 5’-RACE PCR

For rapid amplification of cDNA ends the GeneRacer® Kit with SuperScript® III RT and Zero Blunt® TOPO® PCR Cloning Kit for Sequencing (Invitrogen) were used. The 5’ ends were amplified by nested PCR using HotStar Mastermix (Qiagen) and Phusion Polymerase (Finnzymes). Primers and sequenced clones are available upon request.

### RNA Fluorescence in situ hybridization

RNA detection was performed according to de Planell-Saguer [37]. Specifically, the LNA ISH with following tyramide signal amplification protocol was used. CMK cells were prepared as cytospins from fresh mock cultures. All TAMRA-conjugated LNA probes were designed and synthesized by Exiqon. Fluorescence microscopy was carried out on a BZ9000 (Keyence), data analysis was performed with Biorevo Software (Keyence).

### Statistical analysis

Statistical evaluation between two groups was carried out using Student’s t-test and for more than two groups by 2-way ANOVA with Tukey’s or Sidak’s post-hoc analysis. The level of significance was set at P < 0.05. All data are presented as mean ± s.d. Calculations were performed using GraphPad Prism 6.

## Competing interests

The authors declare that they have no competing interests.

## Authors’ contributions

SE, AS, and FS performed molecular studies, statistical analyses and data interpretation. VRT performed experiments. SE and JHK analyzed and interpreted results, supervised the study and wrote the manuscript. JHK designed the research. DR provided lab space and patient material. All authors read and approved the final manuscript.

## Supplementary Material

Additional file 1: Figure S1 **A)** qRT-PCR of *MIR100HG* in shRNA-transdued CMK cells. **B)** Number of shRNA- or ctrl-transduced K562 cells. **C)** Growth competition assay. The fraction of Cerulean^+^ shRNA-transduced cells at indicated time points of culture is shown in relation to the ctrl construct. Click here for file

Additional file 2: Figure S2 **A)** qRT-PCR of *MONC* in shRNA-transdued CMK cells. **B)** Number of shRNA- or ctrl-transduced K562 cells. **C)** Well pictures of automated microscopy assays in indicated cell lines on day 4 (scale bar: 200 μm) (n = 1). **D)** Number of colonies from methylcellulose-based colony-forming assays of sh-*MONC* transduced K562, CMK and M-07 cells. Click here for file

Additional file 3: Figure S3 Sequence of *MONC* iso-6 transcript (ENST00000445461.2) cloned into LeGO-CeB/lnc vector. Click here for file

Additional file 4: Figure S4 Basal expression levels of *MIR100HG* and *MONC* in CD34^+^ HSPCs compared to CMK cells as determined by qPCR. Click here for file
