# Supplementary figures and images for: LincRNAs MONC and MIR100HG act as oncogenes in acute megakaryoblastic leukemia
Source: Mol Cancer. 2014 Jul 15;13:171. doi: 10.1186/1476-4598-13-171 (PMC4118279; doi:10.1186/1476-4598-13-171)

Figure 1

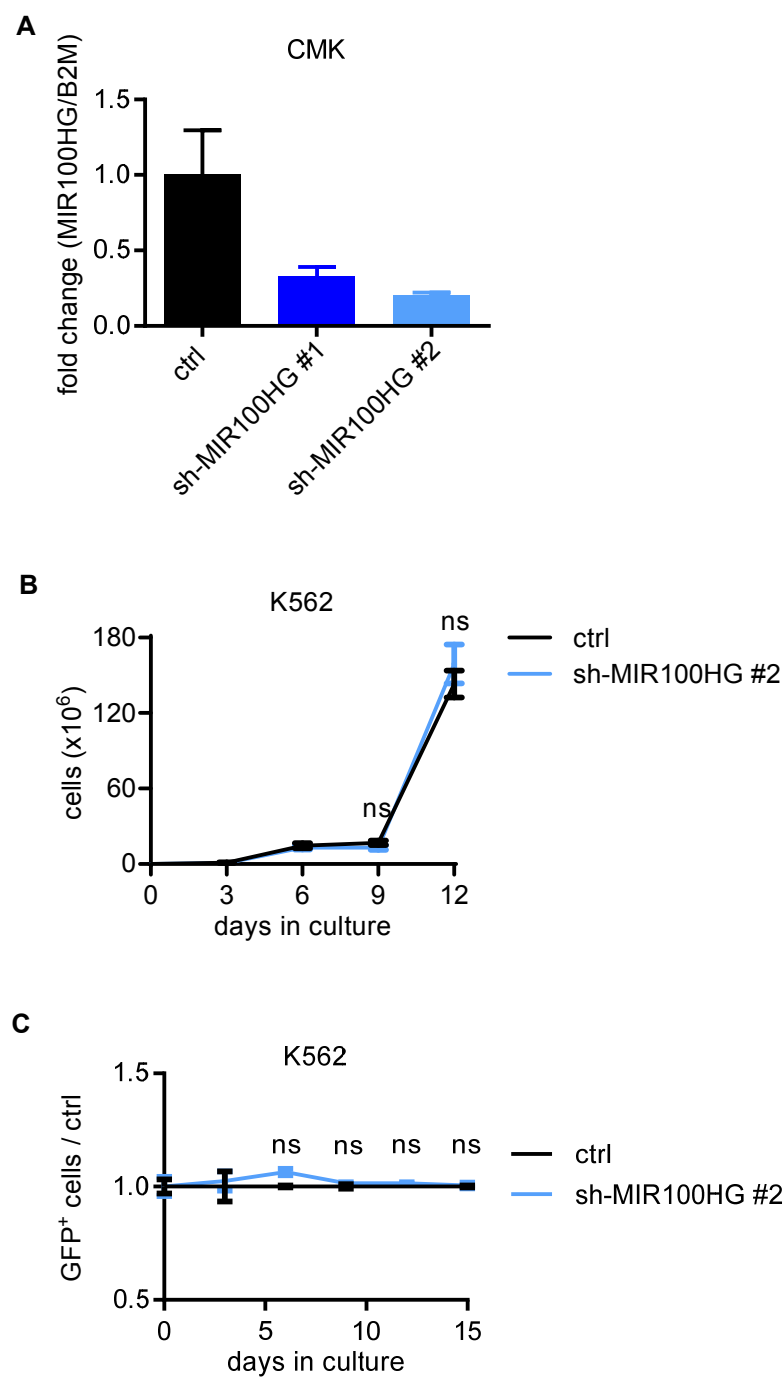

Supplement: Additional file 1: Figure S1 — A) qRT-PCR of MIR100HG in shRNA-transdued CMK cells. B) Number of shRNA- or ctrl-transduced K562 cells. C) Growth competition assay. The fraction of Cerulean+ shRNA-transduced cells at indicated time points of culture is shown in relation to the ctrl construct. [file 1476-4598-13-171-S1.pdf]

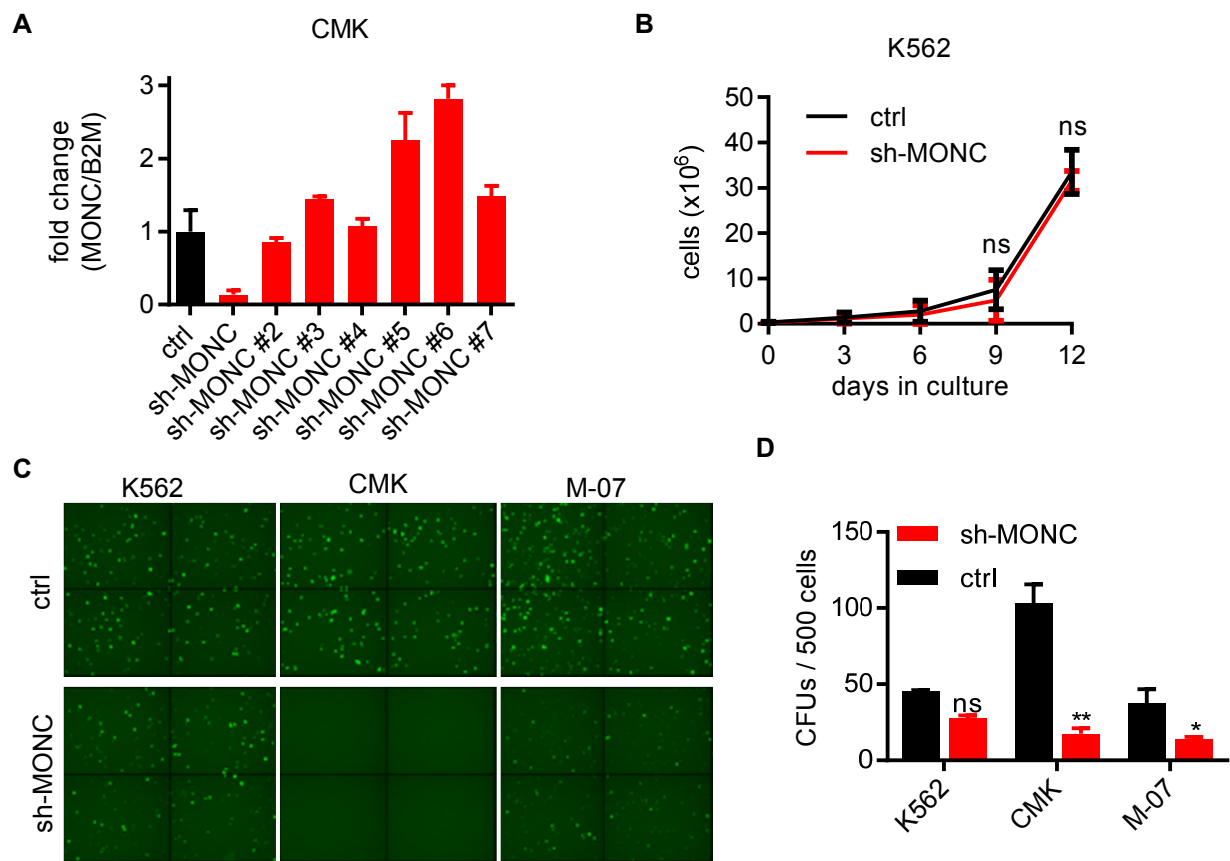

Supplement: Additional file 2: Figure S2 — A) qRT-PCR of MONC in shRNA-transdued CMK cells. B) Number of shRNA- or ctrl-transduced K562 cells. C) Well pictures of automated microscopy assays in indicated cell lines on day 4 (scale bar: 200 μm) (n = 1). D) Number of colonies from methylcellulose-based colony-forming assays of sh-MONC transduced K562, CMK and M-07 cells. [file 1476-4598-13-171-S2.pdf]

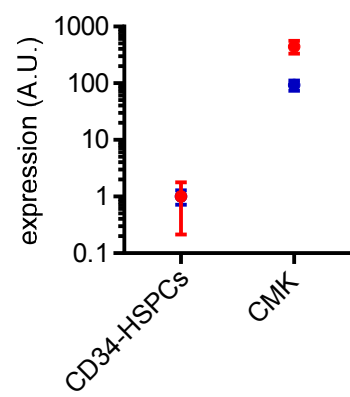

Supplement: Additional file 4: Figure S4 — Basal expression levels of MIR100HG and MONC in CD34+ HSPCs compared to CMK cells as determined by qPCR. [file 1476-4598-13-171-S4.pdf]
