# Supplementary material for: LincRNAs MONC and MIR100HG act as oncogenes in acute megakaryoblastic leukemia
Source: Mol Cancer. 2014 Jul 15;13:171. doi: 10.1186/1476-4598-13-171 (PMC4118279; doi:10.1186/1476-4598-13-171)
Supplement: Additional file 3: Figure S3 — Sequence of MONC iso-6 transcript (ENST00000445461.2) cloned into LeGO-CeB/lnc vector. [file 1476-4598-13-171-S3.pdf]

MONC: ENST00000445461.2

ATCGCCTACTTCAGGCGGTGGCAAGCGTATGGAAATTATATTAGAACTTCAGCAAAGTGC
ACAGCTGCATAATAGAGAAGCCGGCACTCAGATTGACAGGCAAATAAGCCAGCTTGTGGA
AGGGGCCTCTGGGTGGCAGCTCTGGGCTTTTGGGTGTTGGACAACCATAGGCAAAACTGA
AATGGAACAGGGGCAGAAAATAGCACACTTTTACTCTACGCAACTGTTGCTTTTTCATGA
TCTTCAGGAATTTTACTGGGATAATTATCCAAATAAATTGCAAGCATTCTATCCAAATGG
AGCTCTTTCTGAGATGAAGAGAATTCTCAATGTCAAGATTTGAACAAGAAGAGAATGGAA
TACACAATATGGACATCCATAAAAATTCATCAGAGAGCATTTTACTACTGAGCTGCAAAG
GGAAAAACTTAAAATGGATATGAAAAGTGAAGAAAGTGATCATAGGAGAAAACCATTTCA
GATGACAAGAGCACCTCAAAGGCAGCAGCCTCAAGGAGCAGCCATGGCCCCAGACTTGTC
GCACGGATGCAGAAAACTTAATGGAGGAGGCTGAGGTCAGAATGGGAAGAGTTTTTAAAA
AATAAAAAGGGGAGCTAATATGTGAGGAACTGATTGCTGTGCATTATTTTCCACTGAACA
GACCTTCTTTTAACTTCTAATCTCAAGCACATAGCAGCTGTTGTTTTCAT
